# Bariatric surgery in patients with psychiatric comorbidity: Significant weight loss and improvement of physical quality of life

**DOI:** 10.1111/cob.12373

**Published:** 2020-05-18

**Authors:** Karlijn J. Vermeer, Valerie M. Monpellier, Wiepke Cahn, Ignace M. C. Janssen

**Affiliations:** ^1^ Nederlandse Obesitas Kliniek Huis ter Heide The Netherlands; ^2^ Faculty of Psychiatry University Medical Centre Utrecht Utrecht The Netherlands; ^3^ Nederlandse Obesitas Kliniek West Den Haag The Netherlands

**Keywords:** bariatric surgery, health‐related quality of life, mental health, obesity, psychiatric disorder, weight loss

## Abstract

**Background:**

Patients that have psychiatric comorbidity are thought to lose less weight than the general bariatric population and are therefore sometimes denied surgery. However, there is no scientific evidence for this assumption. The aim of this study is to evaluate the weight loss and health‐related quality of life (HRQoL) in patients with psychiatric disorders who undergo bariatric surgery and compare these patients with a general bariatric population.

**Method:**

Patients who underwent bariatric surgery in 2015 were included. Patients who received individual counselling and had a current DSM IV axis 1 or 2 diagnosis were included in the psychiatric group (n = 163), all other patients in the generic group (n = 2362).Weight and HRQoL were assessed before and 12‐, 24‐, 36‐ and 48‐months after surgery. Data was analysed using regression analyses.

**Results:**

The maximum total weight loss (TWL) was 27.4% in the psychiatric group vs 31.0% in the generic group. Difference in %TWL between the psychiatric and generic group was significant from baseline to all follow‐up moments (*P* < .001).

Improvement of PHS was significantly higher in the generic group from baseline to 12‐month (*P* = .002), 24‐month (*P* = .0018), 36‐month (*P* = .025) and 48‐monthfollow‐up (*P* = .003). Change in mental HRQoL was only different comparing baseline to 48‐monthfollow‐up (*P* = .014).

**Conclusion:**

Although weight loss and change in physical HRQoL was lower in patients with pre‐operative psychiatric disorders, results of this group were still excellent. Thus, patients with psychiatric diagnoses benefit greatly from bariatric surgery and these patients should not be denied weight loss surgery.

**Abbreviations**
BMI: body mass index
CI: cognitive impairment
DSM IV: Diagnostic and Statistical Manual of Mental Disorders IV
HRQoL: health‐related quality of life
MHS: mental health summary of RAND‐36
PHS: physical health summary score of RAND‐36
TWL: total weight loss

## 1 BACKGROUND

Bariatric surgery is the most effective treatment for morbid obesity, resulting in weight loss, a reduction or resolution of comorbid conditions like type 2 diabetes, and improvement of health‐related quality of life (HRQoL).^1^, ^2^, ^3^, ^4^, ^5^, ^6^ However, not all patients benefit equally from bariatric surgery. Mental health problems are thought to be indicative of a worse prognosis in terms of weight loss, since such problems could negatively influence post‐operative eating behaviour and compliance.^7^, ^8^, ^9^, ^10^ Furthermore, there is fear of possible worsening of psychological issues after surgery.^10^, ^11^ According to the European guidelines on metabolic and bariatric surgery, psychopathology, such as severe depressions and personality disorders, is therefore considered a contra‐indication for bariatric surgery.^1^

Given that the prevalence of psychiatric disorders is significantly higher in patients with morbid obesity than in the general population, many patients are rejected to undergo bariatric surgery. Among patients presenting for bariatric surgery 27.3% to 41.8% had an Diagnostic and Statistical Manual of Mental Disorders (DSM) IV reported axis 1 disorder (a clinical disorder) and 22% to 24% was diagnosed with an axis 2 disorder (a personality disorder).^2^, ^7^, ^12^, ^13^, ^14^

Previous systemic reviews and a meta‐analysis have attempted to demonstrate an association between psychiatric disorders and weight loss. No evidence was found that patients with pre‐operative psychiatric disorders had significantly less post‐operative weight loss. However, the results from the studies included in these reviews are inconsistent and sometimes show conflicting results. Furthermore, the included studies yielded evidence, which was of moderate quality, partly because the psychiatric disorders were not well‐defined.^2^, ^14^, ^15^

In addition, weight loss is not the only indicator of success after bariatric surgery: HRQoL is considered another primary outcome after bariatric surgery.^16^ Although HRQoL is generally lower in patients with mental health problems, there are indications that weight loss can also reduce psychiatric symptoms in these patients and improve the overall HRQoL.^17^, ^18^ Contrary to this, it was suggested that patients with psychiatric comorbidity might have lower HRQoL after bariatric surgery.^14^

Since there is a high population of patients with extreme obesity and psychiatric disorders who are at risk of developing other severe comorbidities if they remain untreated, further research is needed. In this study, we will assess a large population of patients with psychiatric disorders who have undergone bariatric surgery, focussing on both weight loss and HRQoL after surgery. The aim will be to assess 4‐year weight loss and HRQoL in the total psychiatric population. Second, outcomes of four groups of psychiatric patients will be compared: patients with an axis 1 disorder, patients with an axis 2 disorder, patients with an axis 1 and 2 disorder and patients with cognitive impairment. Third, the results will be compared to the ‘general’ bariatric population.

## 2 METHODS

### 2.1 Standard treatment

All patients included in this study were being treated at the Nederlandse Obesitas Kliniek (NOK, Dutch Obesity Clinic). Treatment at the NOK involves an extensive pre‐ and post‐operative counselling program in addition to the bariatric procedure. The counselling is conducted by an interdisciplinary team, consisting of a medical doctor, psychologist, dietician and physical therapist.^19^ All patients are screened according to the international criteria for bariatric surgery, the IFSO‐criteria, by the interdisciplinary team and subsequently assigned to standard (group) or individual counselling, only after being accounted mentally stable enough.^1^ Most common reasons for patients to receive individual counselling are language barriers, previous bariatric surgical treatment and severe psychiatric comorbid conditions.

### 2.2 Patient and data selection

Patients were selected from a prospective database, provided that they underwent bariatric surgery in 2015. Data was collected up to April 2020. The reason for individual counselling was assessed through screening of the electronic patient record. Only patients who received individual counselling and had a current psychiatric diagnosis or reported a cognitive impairment were included for analysis in the ‘psychiatric group’. All patients who underwent bariatric surgery in the same year who received standard (group‐) counselling were included in the ‘generic group’.

### 2.3 Psychiatric diagnosis

To define the psychiatric diagnosis the following variables were selected from the electronic patient record: reason for individual counselling, past and current psychiatric diagnosis and/or cognitive impairment and the use of psychotropic drugs.

The diagnoses were than grouped into DSM IV axis 1 and axis 2 disorders: clinical disorders vs personality disorders respectively. All patients with current psychiatric disorders on axis 1 and/or axis 2 were assigned to respectively axis 1 group, axis 2 group and axis 1 + 2 group. Although cognitive disorders are strictly taken a subgroup of the DSM IV axis 2, previous investigation has demonstrated that cognitively impaired patients show slightly deviant behaviour.^20^, ^21^, ^22^ Therefore, patients who had no known psychiatric disorder but reported to be cognitively impaired (IQ was not formally assessed in the NOK), were assigned to a fourth group: the cognitive impairment (CI) group.

Patients who were prescribed a psychotropic drug other than minor tranquillizers such as benzodiazepines, but had no known psychiatric diagnosis were also considered to have a current diagnosis on axis 1. Mental health problems requiring psychiatric care were also included. Moreover, patients with personality traits were also included in the axis 2 group.

At the NOK, eating disorders are considered a contra‐indication for bariatric surgery and therefore not included in this analysis.

### 2.4 Body weight and other parameters

Body weight was assessed at pre‐operative screening and 12‐, 24‐, 36‐ and 48‐months after surgery. Height was assessed during pre‐operative screening. Body mass index (BMI in kg/m^2^) and percentage of total weight loss (%TWL) were calculated for baseline (only BMI) and follow‐up. The following parameters were also registered: age, gender, type of surgery and whether this was primary or secondary procedure.

### 2.5 Health‐related quality of life

The perceived HRQoL was evaluated at pre‐operative screening and 12‐, 24‐, 36‐ and 48‐months after surgery using the RAND‐36 questionnaire. The RAND‐36 is one of the most used questionnaires to assess HRQoL in bariatric patients.^16^ It consists of 36 questions and 9 scales and can be used to calculate two subscales: physical health summary (PHS) and mental health summary (MHS). Scores range from 0 to 100, where a higher score represents a higher HRQoL.

### 2.6 Statistical analysis

Continuous variables were visually inspected and tested for normality by the Shapiro‐Wilk test. Descriptive statistics summarized baseline patients' characteristics. Differences in baseline characteristics between the psychiatric and generic group were assessed with *t*‐test and chi‐square test. Subsequently patients were divided in the four psychiatric groups: axis 1 group, axis 2 group, axis 1 + 2 group and CI group. Differences between the groups regarding baseline characteristics were analysed with one‐way ANOVA. These analyses were performed using SPSS (version 24) statistical software.

Then a linear mixed model was conducted to assess how %TWL changed from baseline to 12, 24, 36 and 48 months in the psychiatric group. First, the change of %TWL over all follow‐up moments was assessed with random intercept, thereby the model considers different intercepts for each patient. Then type of surgery, gender, baseline BMI and age were added to the model as fixed effects. In the last part of the model, the differences within the psychiatric groups were assessed (effect modification of the four groups). In a second mixed model, the differences between the psychiatric and generic group were studied. The same model was performed for the changes in the two RAND subtotal scores: the PHS and MHS.

All assumptions for regression analysis were met. These analyses were performed using STATA, version 13 (StataCorp. 2013. Stata 13 Base Reference Manual. College Station, Texas: Stata Press). Findings were considered statistically significant if the *P*‐value was <.05.

## 3 RESULTS

### 3.1 Study population

A total of 163 patients were included in the psychiatric group and 2362 in the generic group. In the psychiatric group, there were significantly less females (66.9% vs 79.5%, *P* < .001). There were also less patients who underwent a primary RYGB (61.3% vs 76.0%, *P* < .001). Mean age and baseline BMI were not significantly different between these groups.

### 3.2 Distribution of psychiatric disorders

In the psychiatric group 58.6% had a psychiatric disorder on axis 1; 6.8% had a disorder on axis 2; 16.7% had diagnoses on both axis 1 and 2; 17.9% presented with a cognitive impairment. The most common clinical disorder was a mood disorder (54.9%), with a prevalence of 63.2% in the axis 1 group and a prevalence of 48.1% in the axis 1 + 2 group (Table 1). Most common personality disorder was a cluster B disorder (9.0%), with a prevalence of 44.4% in the axis 1 + 2 group; whereas the axis 2 group had no prevalence of cluster B disorders.

**TABLE 1:** Distribution of psychiatric disorders at baseline (*n* = 133), presented as percentage (no)

	Axis 1	Axis 2	Axis 1 + 2	Total
Mood disorder	63.2% (60)	0%	48.1% (13)	54.9% (73)
Anxiety disorder	11.6% (11)	0%	11.1% (3)	10.5% (14)
Autism spectrum disorder	11.6% (11)	0%	0%	8.3% (11)
Disorder not otherwise specified/unknown	7.4% (7)	0%	3.7% (1)	6.0% (8)
Post‐traumatic stress disorder	13.7% (13)	0%	18.5% (5)	13.5% (18)
Attention deficit disorder/attention deficit hyperactivity disorder	11.6% (11)	0%	25.9% (7)	13.5% (18)
Remaining clinical disorder	6.3% (6)	0%	7.4% (2)	0%
Suspicion of personality disorder/personality traits	0%	45.5% (5)	29.6% (8)	9.0% (12)
Personality disorder not otherwise specified	0%	27.3% (3)	11.1% (3)	4.5% (6)
Cluster B personality disorder^a^	0%	0%	44.4% (12)	9.0% (12)
Cluster C personality disorder^b^	0%	36.4% (4)	14.8% (4)	6.0% (8)
Cognitive impairment	17.9% (17)	0% (0)	7.4% (2)	14.2% (19)

a Antisocial personality disorder, borderline personality disorder, histrionic personality disorder, narcistic personality disorder.

b Avoidant personality disorder, anxious personality disorder, obsessive compulsive personality disorder.

### 3.3 Weight loss in psychiatric group

**FIGURE 1:**
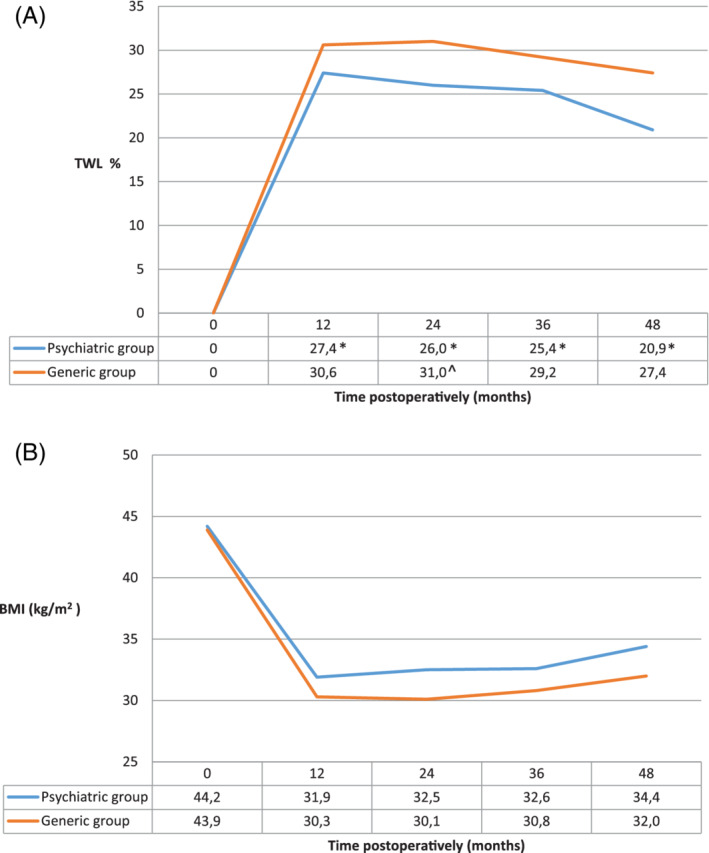
A: Percentage total weight loss (%TWL) in the total psychiatric group compared to the generic population. B: Change in body mass index (BMI, kg/m^2^) in the total psychiatric group compared to the generic population. *Significant difference in score change from baseline to follow‐up moment compared to generic group (*P* < .05); ^**^**^Significant difference in score change from previous follow‐up moment to current follow‐up moment compared to generic group (*P* < .05)

In the psychiatric group, mean BMI was 31.9 kg/m^2^ (±6.1) at 12‐monthfollow‐up and TWL was 27.4% (±10.0) (Figure 1). TWL was 26.0% (±11.8) at 24‐monthfollow‐up, 24.5% (±12.8) at 36 months and 21.6% (±14.3) at 48 months. There was a significant change in %TWL from baseline to all follow‐up moments (*P* < .001 in all). There was no significant difference in %TWL between 12‐ and 24‐monthfollow‐up (*P* = .429) and between 24‐ and 36‐monthfollow‐up (*P* = .234). At 48‐monthfollow‐up, %TWL was significantly lower than at 36 months (*P* < .001).

### 3.4 Psychiatric disorders and weight loss

There were no differences in baseline characteristics between the four groups (Table 2). There were significant differences in %TWL between the four groups with weight loss being lowest in the axis 1 group and highest in the axis 2 group. The axis 2 group had significantly higher %TWL compared to the axis 1 group at 12‐month (*P* = .025), 24‐month (*P* = .001), 36‐month (*P =* .007) and 48‐monthfollow‐up (*P* = .016). At 36‐monthfollow‐up, the %TWL of the axis 1 group was also significantly lower compared to the CI group (*P* = .001); this was the same at 48‐monthfollow‐up in comparison to the axis 1 + 2 group (*P* = .042) and the CI group (*P* = .001).At 24 months and 36 months, the axis 1 + 2 group had significantly lower %TWL then the axis 2 group (*P* = .011 and *P* = .044, respectively).

**TABLE 2:** Baseline characteristics and weight loss results in the psychiatric group, for each of the separate psychiatric disorders

	Axis 1 (n = 95)		Axis 2 (n = 11)		Axis 1 + 2 (n = 27)		Cognitive impairment (n = 30)	
Percentage (no.)								
Female gender	95	68.4% (65)	11	45.5% (5)	27	77.8% (21)	30	60.0% (18)
Primary RYGB	95	57.9% (55)	11	72.7% (8)	27	55.6% (15)	30	73.3% (22)
Mean ± SD								
Age	95	48.0 ± 10.5	11	47.8 ± 13.3	27	45.0 ± 11.4	30	50.2 ± 9.4
BL BMI, kg/m^2^	95	43.3 ± 7.9	11	44.6 ± 7.3	27	45.6 ± 6.9	30	45.8 ± 5.6
12 M BMI, kg/m^2^	86	31.7 ± 6.4	8	30.0 ± 5.3	25	32.8 ± 6.5	29	32.3 ± 5.0
24 M BMI, kg/m^2^	55	32.4 ± 6.4	5	29.5 ± 8.6	17	33.5 ± 6.8	23	32.6 ± 5.5
36 M BMI, kg/m^2^	62	32.7 ± 7.3	5	31.2 ± 8.0	17	33.8 ± 7.5	22	31.6 ± 5.1
48 M BMI, kg/m^2^	43	34.6 ± 8.1	3	34.9 ± 10.3	14	33.7 ± 6.6	19	33.3 ± 6.6
12 M %TWL	86	25.6 ± 10.8 α	8	34.0 ± 5.5	25	28.1 ± 8.7	29	29.5 ± 8.7
24 M %TWL	55	24.1 ± 11.7 α	5	38.7 ± 12.8 β	17	25.2 ± 9.7	23	28.5 ± 11.9
36 M %TWL	62	23.2 ± 13.1 α	5	35.0 ± 10.7 β	17	25.1 ± 11.2	22	29.4 ± 12.4 δ
48 M %TWL	43	18.1 ± 13.6 α	3	30.0 ± 8.0	14	24.1 ± 12.4 δ	19	26.3 ± 14.6 δ

Abbreviations: %TWL, % total weight loss; 12 M, 12 months follow‐up; 24 M, 24 months follow‐up; 36 M, 36 months follow‐up; 48 M, 48 months follow‐up; BL, baseline; BMI, body mass index.

α = significant difference compared to axis 2 group, *P* < .05; β = significant difference compared to axis 1 + 2 group, *P* < .05; δ = significant difference compared to axis 1 group, *P* < .05.

### 3.5 Weight loss: psychiatric vs generic group

Mean BMI in the generic group was 43.9 kg/m^2^ (±5.6) at baseline; TWL was 30.6% (±4.8) after 12 months, 31.0% (±10.1) after 24 months, 29.2% (±9.9) after 36 months and 27.4% (±10.6) after 48 months (Figure 1). Difference in %TWL from baseline to follow‐up was significantly higher in the generic group compared to the psychiatric group at all follow‐up moments (*P* < .001 in all) and when comparing 12‐ with 24‐monthfollow‐up (*P* = .022). There was no significant difference between 24‐ and 36‐month (*P* = .184) and 36‐ and 48‐month (*P* = .097).

### 3.6 HRQoL in psychiatric group

**FIGURE 2:**
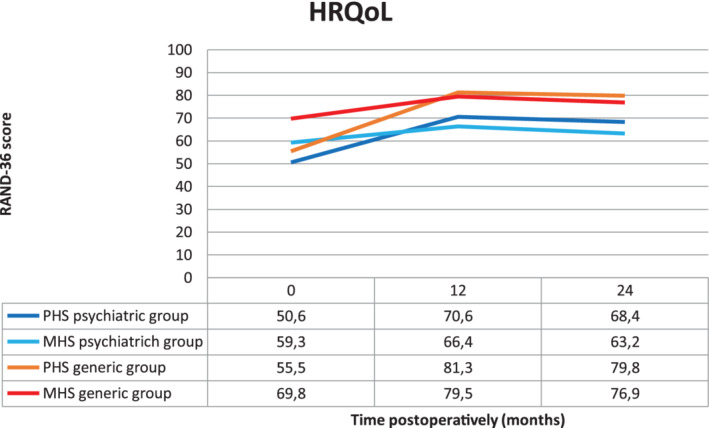
RAND‐36 physical health summary (PHS) and mental health summary (MHS) scores in the psychiatric group compared to the generic group. *Significant difference compared to generic group (*P* < .05)

The PHS significantly improved when comparing baseline to all follow‐up moments (*P* in all <.001) (Figure 2). There was no significant difference in PHS between 12‐ and 24‐monthfollow‐up (*P* = .249), between 24‐ and 36‐monthfollow‐up (*P* = .566) and between 36‐ and 48‐monthfollow‐up (*P* = .359).

The MHS significantly improved from baseline to 12‐monthfollow‐up (*P* = .003); after that there was a decline. There were no statistically significant differences between baseline and 24‐month (*P* = .162), 36‐month (*P* = .651) and 48‐month (*P* = .249) follow‐up. There were also no differences between the follow‐up moments: 12‐ vs 24‐month (*P* = .234), 24‐ vs 36‐month (*P* = .092) and 36‐ vs 48‐month (*P* = .491).

### 3.7 Psychiatric disorders and HRQoL

There were no differences between the psychiatric groups in the change of PHS at any of the follow‐up moments (Table 3). Change in MHS scores at 12 months differed significantly between the axis 1 and axis 2 group (*P* = .018) and between axis 1 + 2 and axis 2 group at 12 months (*P* = .006).

**TABLE 3:** RAND‐36 scores in the psychiatric group, for each of the separate psychiatric disorders, presented as mean ± SD

	Axis 1 (n = 95)		Axis 2 (n = 11)		Axis 1 + 2 (n = 27)		Cognitive impairment (n = 30)	
Physical health summary								
BL	83	49.6 ± 22.2	9	46.3 ± 28.8	24	47.2 ± 23.3	25	58.9 ± 21.5
12 M	63	68.9 ± 22.5	6	79.0 ± 23.6	22	62.4 ± 24.9	26	79.8 ± 21.5
24 M	42	66.0 ± 23.1	4	68.0 ± 41.0	14	70.7 ± 25.0	20	72.0 ± 19.7
36 M	47	66.6 ± 26.0	5	68.6 ± 35.1	16	57.5 ± 28.5	18	76.6 ± 21.9
48 M	36	60.4 ± 24.8	4	60.6 ± 40.8	13	56.8 ± 29.0	16	73.0 ± 22.4
Mental health summary								
BL	83	60.0 ± 22.1	9	53.7 ± 25.7	24	54.0 ± 21.3	25	64.2 ± 17.9
12 M	63	66.2 ± 23.1 α	6	81.9 ± 16.4 β	22	53.0 ± 28.8	26	74.6 ± 22.6
24 M	42	61.6 ± 25.9	4	66.7 ± 37.6	14	59.5 ± 25.4	20	68.3 ± 20.4
36 M	47	58.8 ± 27.8	5	67.4 ± 32.2	16	49.1 ± 27.5	18	70.3 ± 19.4
48 M	36	57.6 ± 24.4	4	57.7 ± 32.5	13	42.6 ± 21.5	16	61.7 ± 25.9

Abbreviations: BL, baseline; %TWL, % total weight loss; 12 M, 12 months follow‐up; 24 M, 24 months follow‐up; 36 M, 36 months follow‐up; 48 M, 48 months follow‐up.

α = significant difference compared to axis 2 group, *P* < .05; β = significant difference compared to axis 1 + 2 group, *P* < .05.

### 3.8 HRQoL: psychiatric vs generic group

Change in PHS was significantly different when comparing the psychiatric and generic group from baseline to follow‐up: compared to 12‐monthfollow‐up (*P* = .002), to 24‐monthfollow‐up (*P* = .0018), to 36‐monthfollow‐up (*P* = .025) and to 48‐monthfollow‐up (*P* = .003) (Figure 2). There were no differences comparing the other follow‐up moments.

Change in MHS was only significantly different when comparing the psychiatric and generic group from baseline to 48‐monthfollow‐up (*P* = .014). There were no significant differences between the other follow‐up moments.

## 4 DISCUSSION

The aim of this study was to investigate the weight loss and HRQoL of patients with psychiatric comorbidity following bariatric surgery and compare the results with a generic bariatric population. The results show that patients with psychiatric disorders had an average maximum TWL of 27.4% 1 year after surgery. This TWL declined to 20.9% 4 years after surgery. This was still only about 5% less than the generic bariatric patient. Patients in the axis 1 group seemed to have the lowest weight loss, especially at mid‐termfollow‐up.

Although change in physical HRQoL after surgery was significantly higher in the generic group, physical HRQoL still improved significantly in the psychiatric group. Mental HRQoL improved during the first 12 months and then stabilized in both the psychiatric and generic group. Although, 4 years after surgery the MHS was 4 points lower than at baseline, while in the generic group it was 3 points higher. The axis 2 group demonstrated a greater change in HRQoL than the axis 1 and CI group. Thus, bariatric surgery seems to be an effective treatment for morbid obesity, also in patients with psychiatric disorders.

Three large meta‐analysis concluded that there was no difference in weight when comparing patients with and with psychiatric comorbidity.^2^, ^14^, ^15^ This study contradicts these results, since a significant difference was found between the psychiatric group and the ‘general’ bariatric population. However, the 5% lower weight loss in our study should not be the reason to deny patients with psychiatric comorbidity bariatric surgery. Especially since previous studies have shown that even a 5% reduction of body weight in patients with obesity can already lead to great health improvements, like lower cholesterol levels, decreased blood pressure and improved beta cell function and insulin sensitivity.^23^, ^24^ In addition, a TWL above 20% has previously been described a successful result. The average TWL of 20.9% after 4 years in the psychiatric group is therefore considered sufficient.^23^

In our study, post‐operative weight loss seems to be different between the psychiatric subgroups. Previous studies could not relate axis 1 disturbances, like depression and anxiety, to lower weight loss.^2^, ^14^, ^15^, ^25^ In our study however, patients with an axis 1 diagnosis, which were mostly anxiety and depressive disorders, had the lowest weight loss (16.8% after 4 years) of all four groups. It could be that in our group the axis 1 disorders were more serious, since patients with mild axis 1 disorders are mostly treated in group counselling.

Surprisingly, weight loss was very high in patients with an axis 2 disorder, TWL was 30.0%, while other studies showed a negative effect of personality disorders.^14^, ^15^ This might be explained by the fact that only specific personality disorders seem to interfere with weight loss.^26^ Moreover, patients in this study had a strict multidisciplinary program before and after surgery, which can be a reason for improved weight loss results. However, the current results might also be explained by the relatively small number of patients and low follow‐up rate in the axis 2 group, with only three patients left after 4 years of follow‐up. As a result, it remains difficult to draw conclusions for this subgroup.

Another interesting finding was that patients with a cognitive impairment demonstrated relatively high weight loss, which was significantly better than the axis 1 group and almost the same as the generic group. Possibly psychotropic medication, which is only prescribed to patients with psychiatric disorders and not to patients who are solely cognitively impaired, might limit weight loss. In addition, the NOK provides structured care and demands that patients have a well‐functioning support system involved in the care program. This might also explain that the supposed deficits in executive function and memory in these patients do not limit their weight loss as much as one might expect.

Physical HRQoL improved in all psychiatric patients, with no differences between the groups. Even though actual scores were higher in the generic group, there were no differences between the psychiatric and generic patients when comparing changes between follow‐up moments. Therefore, the changes in physical HRQoL after surgery were comparable between the psychiatric group and the generic group.

In the psychiatric group, mental HRQoL only improved up to 12 months and then decreased. At 4‐yearfollow‐up, the mean score was 3.5 point below the MHS score before surgery. It seems that the improvement in mental HRQoL in the psychiatric group is only temporarily. In the generic group mental HRQoL also declined after 1‐yearfollow‐up, but these changes were less than in the psychiatric group. This might point out that other factors than BMI contribute significantly to the mental HRQoL in patients with psychiatric comorbidity. The fact that greater improvements were found for physical HRQoL could support this: by losing weight, the psychiatric symptoms might reduce but not remit completely. Furthermore, the wide distribution of the PHS and MHS indicates that even within the subgroups, patients apparently perceive their HRQoL very different. In addition, the RAND‐36 provides information on the mental state of the patients, but it is no validated tool to assess the state of psychiatric disorders. For future research in this specific population, it is therefore essential to use a tool specifically designed to evaluate mental health problems. This could provide further insight in the decline in mental state in long term, as demonstrated in this research.

Our study has some limitations. First, this is a retrospective study and therefore the main limitation is the assessment of psychiatric diagnosis using the electronic patient record. Second, only patients receiving individual counselling were assessed for inclusion in the psychiatric group. As a consequence, it is possible that patients assigned to the generic group could have also suffered from a (less severe) psychiatric disorder. Third, there was a risk of selection bias as patients who suffered from severe mental health problems, and therefore considered not psychiatrically stable, were not operated and therefore not included. Finally, 48‐months compliance was 48.5%, this is a known problem in bariatric patients. This is in concordance with previous research, but by conducting a mixed model analysis, we attempted to limit study confounding.^27^ However, since this low compliance especially resulted in low numbers of patients in the different groups, we cannot draw conclusions when comparing the groups.

## 5 CONCLUSION

The current European guideline on bariatric surgery considers non‐stabilized psychotic disorders, severe depression and personality disorders to be a contra‐indication, unless specifically advised by a psychiatrist experienced in obesity.^1^ Based on our findings, it might be too early to update the guideline, since we do not have knowledge about the risk of worsening of psychiatric symptoms after the surgery. However, our findings show that patients with psychiatric disorders should not be excluded from surgery as they show significant weight loss and improvement of physical HRQoL. Given that the health risks related to morbid obesity are severe, physicians should carefully asses a patient before deciding to reject a candidate on mental health grounds. A structured, multidisciplinary perioperative counselling program for this group of patients seems to improve the success of the intervention.

## CONFLICT OF INTEREST

V. M. M. works as a researcher at the clinic were the research was conducted and I. M. C. J. is the central medical officer of this clinic. The other authors have no competing interest to declare.

## AUTHOR CONTRIBUTIONS

Study concept and design: V. M. M., W. C. and I. M. C. J. Acquisition of data: K. J. V. and V. M. M. Analysis and interpretation of data: K. J. V. and V. M. M. Drafting of manuscript: K. J. V. Critical revision of the manuscript for important intellectual content: V. M. M., W. C. and I. M. C. J. Final approval of the submitted version: V. M. M., W. C. and I. M. C. J. Administrative, technical or material support: K. J. V. and V. M. M.

## ETHICS STATEMENT

All procedures performed in studies involving human participants were in accordance with the ethical standards of the institutional and/or national research committee and with the 1964 Helsinki declaration and its later amendments or comparable ethical standards.

For this type of study, formal consent is not required.

## Data Availability

The data are not publicly available due to privacy restrictions, but are available from the author (V. M. M.) on reasonable request.
